# 

**Published:** 1874-01

**Authors:** 


### TABLE OF CONTENTS.

ORIGIAL COMMUNICATIONS.

Ophthalmia Neonatorum. By J. C. Bishop, M.D. . . . . . . . 1
Infant Management. By F. K. Bailey, M.D. . . . . . . . 7
Case of Cancer of the Pancreas and Modified Transfusion of Blood. By Ephriam Cutter, M.D. . . . . . . . . . . . . 12
A Law of Registration of Births, Marriages and Deaths. By C. B. Nottingham, M. D. - - - - - - - - - - - - 16
On the Use of Veratrum Viride in Pneumonia and Certain Forms of Fever. By Wm. L. M. Harris, M.D. . . . . . . . . . . . . 20

BREVITIES.

Ossification of the Placenta; Hypophosphite of Soda and Quinine in Enlargement of the Spleen—23. For Diphtheria; Fluid Extract Phytolacca Decandra—24. What Was It; Position in Labor—25. Chloral in Cholera Morbus; Magnesia as a Surgical Dressing—26.

EDITORIAL EXTRACTS AND GLEANINGS.

Nævus Treated by Croton Oil; Ligation of the Brachial Artery—Secondary Hemorrhage on the Forty-fourth Day; Persistence of Hymen; Nitric Acid in the Treatment of Uterine Diseases; Phosphorus Mixture; Food for Infants: Encysted Tumor of the Neck; Frequently Recurring Epistaxis Relieved by Ergot after Local Treatment had Failed. By T. Curtis Smith, M,D. 27
Influence of School Life on Vision; New Incision for Linear and Traction of Cataract; Destruction of the Eye by Herpes Zoster Ophthalmicus; Examination of Nasal and Aural Cavities and Application to Them; Episcloral Melanotic Sarcoma; Night Sweats. By Swan M. Burnett, M.D. . 31
Treatment of Gastralgia; Atropia in Salivation; Yellow Fever in New Orleans, 1873; Puerperal Amaurosis—Its Importance as a Symptom; Remarkable Mode of Infanticide; Chloral in Asthma; A Vacancy for a Doctor; A Post-obit; Quinine During Pregnancy; Belladonna Plaster in Obstinate Vomiting and Sea-Sickness; For Cholera. By A. R. Kilpatrick, M.D. 36
Pancreatine; A Test for Morphia; How to Distinguish Sulphate of Morphine fram Quinine; Ergot in Headache; Pills of Sulphate of Quinia; Transfusion of Blood. By R. C. Word, M.D. . . . . . . . . . 41

MISCELLANEOUS GLANINGS FROM EXCHANGES.

Risks Attending the Use of Bromide of Patassium; Treatment of Inflamed Hæmorrhoids in Parturient Women—46. Local Employment of Chlorate of Potash in Cancerous Sores; Night Sweats of Phthisis; Amaurosis Cured by the Extraction of Fangs—47. Valerian in Diabetes; Treatment of Cancrum Oris; Belladonna as a Propylactic in Scarlatina—48. Syphilitic Onychia Treated with Acid Nitrate of Mercury; Delirium Tremens at Rosevelt Hospital; Treatment of Consumption at the Brompton Hospital; Removal of Ink Spots—49. Treatment of Diabetes by Arsenic; A Useful Soap; Rest in the Treatment of Consumption of the Lungs; Anti-Neuralgic Snuff—50. Ether vs. Chloroform; Good Effects of Aconite in Acute Pneumonia; To Keep Gum Arabic from Moulding; Treatment of Chronic Eczema of the Genitals; Cerebro-Spinal Fever or Tetanus—51. Treatment of Obstinate Forms of Epilepsy; Treatment of Narcosis of Chloroform; Ergot in Dysentery; On the Prevention of Pitting After Small-Pox—25.

EDITORIAL AND MISCELLANEOUS.

Notices; Premiums, . . . . . . . . . . . . . . 53
Salutatorios, . . . . . . . . . . . . . . . 54
Our Editorial Staff, . . . . . . . . . . . . . 57
Not Responsible; Communications, . . . . . . . . . . 58
Death of Prof. Agassiz, . . . . . . . . . . . . . 59
Death of Dr. S. W. Butler; Our Medical Recruits; We Refer . . . . 60
Crowded; The Toner Dectures; Give them a Word, . . . . . . 61
List of Cash Payments to the Record, . . . . . . . . . . 62

